# Severe hypertension and (renal) thrombotic microangiopathy: solving the puzzle

**DOI:** 10.1007/s40620-023-01659-z

**Published:** 2023-05-31

**Authors:** Florian Wehrmann, Anke von Bergwelt-Baildon, Ulf Schönermarck

**Affiliations:** Department of Medicine IV, University Hospital, LMU Munich, Marchioninistrasse 15, 81377 Munich, Germany

The occurrence of acute hypertension-mediated target organ damage, i.e., to the heart, retina, brain, and/or kidney, in patients with significantly elevated blood pressure (systolic pressure ≥ 180 mmHg and/or diastolic pressure ≥ 120 mmHg) is the defining feature of severe hypertension, which is also called malignant hypertension, or hypertensive emergency [1]. Patients with severe hypertension can present with systemic signs of thrombotic microangiopathy (TMA), i.e., thrombocytopenia and microangiopathic hemolytic anemia, and/or histological signs of TMA in kidney biopsies. Determining the underlying disease causing TMA is essential for selection of the optimal therapy.

However, diagnosis and classification of TMA and especially of patients with atypical hemolytic uremic syndrome (aHUS) is challenging and the underlying etiology is often unknown at initial presentation. While severe hypertension is highly prevalent in patients with aHUS, only 5–15% of patients with malignant hypertension will develop TMA [2, 3] and among the latter, glomerulonephritis is a possible underlying cause [3]. Thus, aHUS remains a fairly rare cause of malignant hypertension. Furthermore, renal-limited TMA without systemic signs of hemolysis and thrombocytopenia can be diagnostically challenging and often results in delaying appropriate therapies.

In the complex pathogenesis of hypertension and TMA, interaction of different driving forces, such as shear stress, endothelial injury and dysfunction, inflammation, oxidative stress and complement-activation, finally leads to platelet aggregation and microvascular thrombosis within the kidney [2, 4, 5]. In primary aHUS (also called complement-mediated TMA [CM-TMA]), dysregulation of the alternative pathway is the driving force to endothelial damage and has been associated with genetic susceptibility factors related to the complement system. In adults, autoantibodies against complement factor H are less frequently involved. aHUS likely results from interactions between genetic susceptibility factors in the complement system and environmental factors (e.g., infections, pregnancy, or injuries) that trigger complement activation and/or endothelial cell damage (so-called complement-amplifying conditions) [2, 6]. In contrast, local complement activation by tissue injury and inflammation can be seen as a consequence of hypertension itself [5]. Such activation might be transient and self-remitting, either spontaneously or under antihypertensive therapy.

To date, primary aHUS remains a diagnosis of exclusion due to the lack of specific diagnostic tests. In contrast, severe hypertension-associated TMA has been included in the spectrum of secondary TMA. However, there is large clinical overlap between both disease entities. Recently, several studies have investigated patients presenting with severe hypertension and TMA on kidney biopsies using clinical, histological and/or genetic data to differentiate between both disease entities [7–9]. The study by Chen et al. in a Chinese patient population comparing 45 patients with severe hypertension and TMA in kidney biopsy (CM-TMA, *n* = 5; severe hypertension-associated TMA, *n* = 40) [10] adds further data.

In the setting of TMA and severe hypertension, some clinical and histological features might help to suggest hypertension rather than primary aHUS as the underlying cause: male gender; age > 45 years; history of hypertension and/or antihypertensive treatment cessation; rapid (< 72 h) resolution of hematological TMA features after strict control of hypertension; left ventricular hypertrophy; no requirement of dialysis [2]. In kidney biopsies with features of TMA the absence of glomerular thrombi might help to exclude CM-TMA [2]. In the study by Chen et al. patients with severe hypertension-associated TMA had significantly more severe left ventricular hypertrophy, less often systemic signs of TMA and less severe kidney failure, but none of these parameters was sufficient to distinguish between the disease entities. The presence of acute glomerular TMA features and arteriolar thrombosis increased the probability of the diagnosis of CM-TMA, but could not distinguish both groups exclusively [10].

When primary aHUS is associated with genetic susceptibility in the complement system, can genetics help to distinguish between primary and secondary TMA forms? Clearly, the detection of pathogenic rare variants occurring significantly more often in affected individuals than healthy individuals can be linked with the diagnosis of CM-TMA. These include loss-of-function variants in complement factor H, complement factor I, membrane cofactor protein and CFH-hybrid genes (e.g., CFHR1-CFH), as well as gain-of-function variants in complement 3 and complement factor B [2, 6].

However, up to 50% of patients clinically diagnosed with primary aHUS do not carry complement gene variants. The penetrance of the disease is highly variable, not all carriers of complement gene variants within families with affected patients will develop aHUS [2, 6]. Moreover, not all detected complement gene variants have documented pathogenic effects. These “variants of unknown or uncertain significance” may contribute to the development of TMA, but their relevance remains to be fully established.

Complement genetics is even more complex. Certain complement gene haplotypes or polymorphisms are classified as risk haplotypes as they are associated with the development of aHUS [2, 6]. They might cause only subtle changes in complement factor activity [6]. However, as common genetic variants they are also highly prevalent in the general population. The existence of protective variants has been described as well [6]. The presence of one or more risk haplotypes has been shown to increase disease penetrance in carriers of pathogenic complement variants in a dose-dependent manner [11].

The accumulation of common variants and/or polymorphisms within several complement genes and potentially even in genes outside the complement system involving vascular physiology and repair might increase the predisposing risk. However, even with accumulation of multiple genetic risk factors a trigger is required to unmask the complement defect. With stronger genetic risk factors only minor external trigger factors are needed, with less severe genetic risk factors stronger external triggers are essential to activate the complement system and trigger a TMA episode.

Recently, several studies have investigated the contribution of complement genetics in severe hypertension-associated TMA. The study of Chen et al. adds further on this topic [10]. The detection of pathogenic rare complement gene variants ranged from 0% in two studies (100 patients, Larsen et al. 2018; 40 patients, Chen et al. 2022) to 36% (26 patients, Timmermans et al. 2020) and 40% (20 patients, Zhang et al. 2021) [6–10]. This difference likely results from differences in patient selection as there is no specific diagnostic test to clearly distinguish patients with primary aHUS. The two studies which more rigorously excluded patients with assumed primary aHUS also reported the frequency of variants of uncertain significance in complement and coagulation genes. Interestingly, they found a high percentage of patients carrying one or more of these variants (40% in the study by Larsen et al., 85% in the study by Chen et al.) [7, 10]. It should be acknowledged that even in these comprehensive studies not all known risk haplotypes (e.g., CFHR copy-number-variations) were studied.

At present, the relevance of common risk haplotypes and variants in complement and coagulation genes for the development of TMA in the circumstances of severe hypertension is unknown but deserves further investigation. Even when postulating the importance of the complement system in severe hypertension-associated TMA the role of complement inhibition is unclear due to the lack of prospective studies, although retrospective case series suggest a benefit of treatment with eculizumab [4].

In routine clinical practice it remains challenging to distinguish primary aHUS (CM-TMA) from severe hypertension-associated TMA due to overlapping clinical presentations and the lack of diagnostic tests. Early implementation of therapeutic complement inhibition is warranted in CM-TMA and enables better kidney survival [12]. In patients with severe hypertension, TMA and severe kidney disease, early complement inhibition should be considered when CM-TMA remains among the possible differential diagnoses, especially with ongoing TMA despite adequate blood pressure control, with progressive deterioration in kidney function, or a family history of TMA [4, 5]. In the absence of a quick, reliable and specific test able to define in vivo activation of the alternative complement pathway, clinicians mainly rely on their clinical experience. Prospective trials are warranted to investigate therapeutic complement inhibition in patients presenting with hypertensive emergency, TMA and severe kidney disease.

## Data Availability

Data sharing not applicable to this article as no datasets were generated or analyzed during the current study.
